# Congenital Pseudoarthrosis of the Tibia: A Narrative Review

**DOI:** 10.7759/cureus.32501

**Published:** 2022-12-14

**Authors:** Hansika Banchhor, Vilas Chimurkar

**Affiliations:** 1 Department of Medical Education, Jawaharlal Nehru Medical College, Datta Meghe Institute of Medical Sciences, Wardha, IND; 2 Department of Anatomy, Jawaharlal Nehru Medical College, Datta Meghe Institute of Medical Sciences, Wardha, IND

**Keywords:** fracture, ilizarov fixation, congenital pseudoarthrosis of the tibia, osteogenesis, neurofibromatosis, cpt

## Abstract

Congenital pseudoarthrosis of the tibia, also known as CPT, is a rare bone disease mostly occurring in the pediatric age group of 10. The case generally represents anterolateral bowing of the tibia with refractures. This condition is quite rare and has very few cases per year. CPT is still a challenging concern in orthopedics. Bone reunion often requires repeated surgical measurements. With advancements, we have new treatment plans that include induced membrane techniques and bone marrow stromal cell grafts of various induced and morphogenetic protein, which still requires confirmation. Different treatment strategies based on surgical, mechanical, and biological concepts have been shown with varying success rates. Ilizarov technique and vascularised fibular grafts have vastly increased the effectiveness in treating CPT of the tibia. Constant and recurrent refracture, residual deformities, and difficulty obtaining bone union remain the challenges in managing CPT. Hence, phasing CPT as bowing the tibia in an anterolateral fashion is more appropriate because it’s a heterogeneous entity with varying prognoses.

## Introduction and background

Congenital pseudoarthrosis of the tibia (CPT) is one of the rare, perplexing, and challenging orthopedic disorders. Its clinical presentations range from extensive complete non-union of bone to simple anterolateral tibial angulation. Classifications of radiographic findings consist of hypertrophic or atrophic pseudarthrosis and dystrophic or cystic lesions [1]. CPT is a congenital, difficult, with less occurrence bone disorder in a population including neonates. The exosomes extracted from the serum of patients affected by CPT inhibit bone formation [2]. It includes challenging complications involving refracture, non-union of the tibia, and failed surgery [3]. The pathophysiology demonstrates highly elevated osteoclasis by the fibrous hamartoma around it and the low osteogenesis and bone morphogenetic protein. This ultimately results in frequent and eventual tibial fractures, atrophy of the bone, and loss of remodeling potential [4]. Although this rare disease, CPT is common and often seen among other arthrosis. Neurofibromatosis type 1, in many cases, is linked with CPT. Anterolateral bowing of the tibia results in pathologic fracture remarks in CPT [5]. CPT is of different types. One is type II, with more occurrence and the poorest prognosis. The common cause of failure in these patients is osteolytic fibromatosis reoccurring [6]. Refracture up to skeletal maturity, the dysplastic segments inhibit soft healing power, difficulty stabilizing small osteoporotic bone fragments in children, and refracture to skeletal maturity are the main issues of concern [7]. The persistent malalignment of the tibia or fibula increases stress on a dystrophic bone after healing, causing refracture [8]. There are various controversies regarding surgery to treat CPT. Some European multicentre studies on CPT of the tibia prove that surgery should be avoided after age three, preferably deferred until age five, because the results were not favored [9]. Currently, the age of surgery for CPT remains controversial and is studied by many [9]. Therapeutic challenge and diagnosis of CPT of the tibia in the late-onset differentiate it from other rare bone deformities. None of the surgical methods is proven superior. But using Ilizarov and reconstruction is a safe, practical, and effective way to deal with it [10]. The reasons for these controversies and conflicts results relate to the form of treatment undertaken. Surgeons worldwide used intramedullary stabilization in addition to the Ilizarov technique with both periosteal and cortical bone grafting and stood with positive results.

## Review

Methodologies

This review involves data collected from various studies, and some of the references have been taken from articles published in journals like PubMed, Scopus, and Web of Science and utilized the keywords to sensitize or search: congenital pseudoarthrosis of the tibia, Ilizarov fixation in congenital pseudoarthrosis of the tibia, management of pseudoarthrosis of the tibia, pathology of pseudoarthrosis of tibia. We excluded the article that was incomplete, not in the English language, or duplicate. Studies have been made on patients presenting with CPT.

Etiology of CPT

The causes of CPT are unknown. Still, certain factors responsible for it are CPT and neuro-fibromatosis seem to relate, at the time of birth or intrauterine trauma and generalized metabolic disturbances [11].

Pathogenesis

The tibia having pseudoarthrosis exhibits insufficient osteogenetic capability followed by mechanical strength. An abnormal fibrovascular tissue mass having a highly cellular nature may result in development in the body cortex. Osteoclastic bone resorption that lacks normal bone modeling gets promoted as it encroaches on the bone cortex. Simultaneously reactional changes occur in the medullary aspect, which leads to vascularization and causes decreased osteogenic capabilities. The similarities in radiological findings in skin neurofibromas of patients having neurofibromatosis and abnormal periosteum may indicate some pathogenic associations between diseases [12].

Pathology

**Figure 1:**
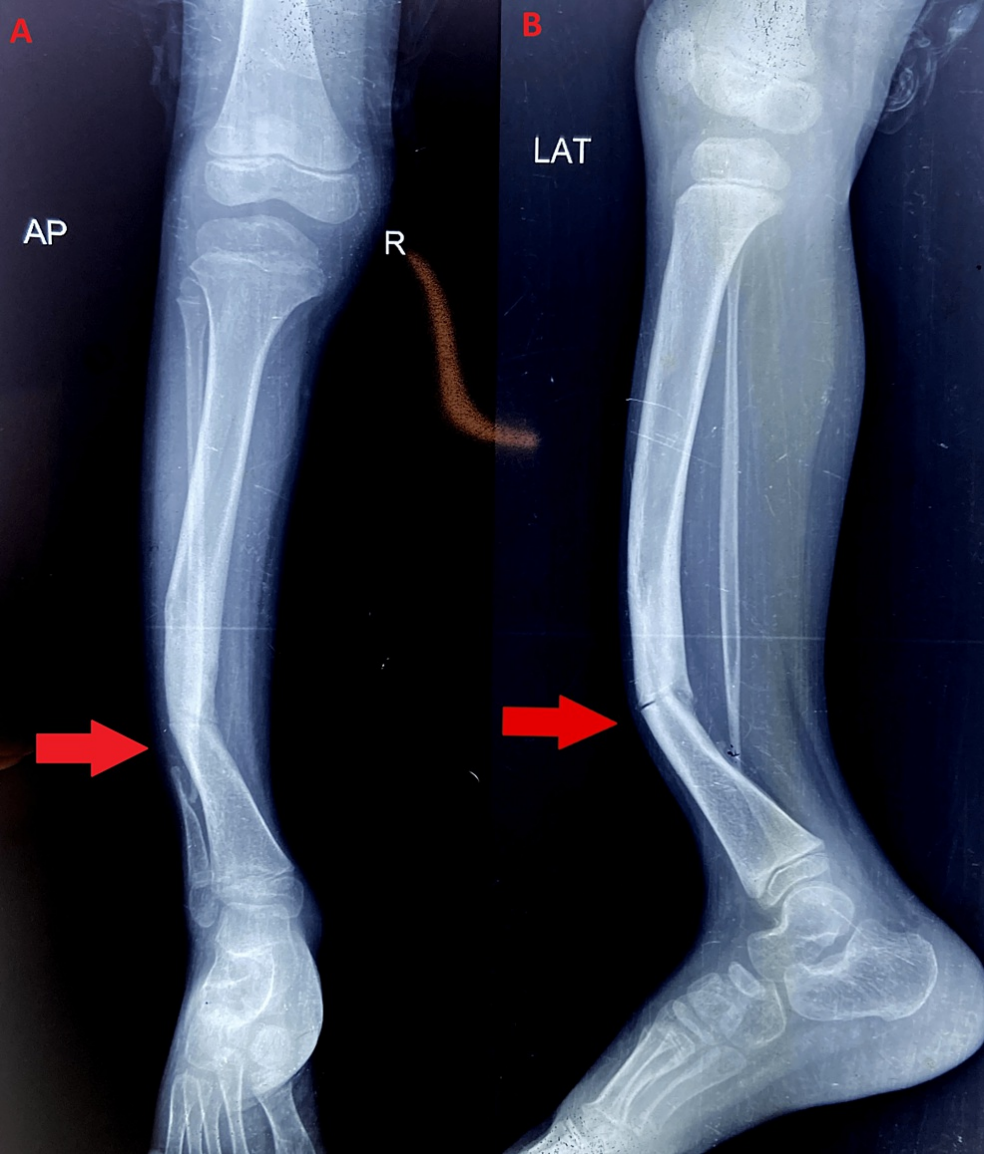
Radiograph showing anterolateral bowing of the tibia. Anterior-posterior and lateral radiographs that were taken before surgery demonstrating anterolateral bowing of the tibia which is the characteristic feature of congenital pseudoarthrosis of the tibia. The author has self-captured.

The main histopathological change is a paucity of vascular ingrowth and highly cellular tissue accompanied by the paucity of vascular ingrowth. Soft tissue can be fibrous tissue, fibrocartilage, or even hyaline cartilage at the pseudoarthrosis site. Figure 1 shows the anterolateral bowing of the tibia.

Diagnostic marker and test

MSC (mesenchymal stem cell) phenotypic characterization in the fifth passage, the MSCs were taken, and preparations were made in the following steps. (1) At 37 °C for a continuous 5 min, cells are treated with trypsin to make cell separate as they adhere. Then they are washed with phosphate-buffered saline and stained with a human analysis kit. (2) As instructed by the company, fluorescence antibody cocktails have negative markers, which include CD34, CD11b, CD19, CD45, and HLA-DR; also, the positive attributes include (CD73, CD90, and CD105) [9]. Senescence assayas per the company-instructed protocol, the percentage of senescent cells under an inverted microscope is observed under 100X magnification in five areas of view and studied by the senescence cells histochemical staining kit [12]. A differentiation assay for confirming the MSC plasticity differentiation assay was conducted. During the fifth passage, cells were harvested and cultured in the medium for chondrogenic, adipogenic, and osteogenic differentiation. Adipogenic and osteogenic potencies are seen by culturing the cells in stempro osteogenesis and adipogenesis. Differentiation has adipogenic and osteogenic potencies. To induce chondrogenic differentiation, under normoxia conditions, at 37 °C, incubation is done, and cells are viewed and observed after 21 days of culturing. Further, it is stained and analyzed for its capacity for osteogenesis, adipogenesis, and chondrogenesis. Stained cells are observed under 100X magnification [13,14].

Type of CPT

Broadly CPT occurs in two types trophic and atrophic. Out of them, atrophic is more common in the human population. Trophic includes cystic where diameter not affected, the distal part of the third tibia represents a cyst, pseudoarthrosis in the fibula may be present, content resembles that of fibrous dysplasia, pseudoarthrosis at different sites like the femur, humerus, clavicle, and ulna. Atrophic coincides with anteriority bowing of the tibia, at the early phase of childhood or at the time of birth, the tibia is represented by narrowed diameter, tibia with complete obliteration in marrow or severe sclerosis, thick periosteum is also differentiating feature, at the site of the fracture, the proliferation of hamartomatous tissue, other bones like fibula also have a chance of fracture, following a fracture, bone end taper. Pseudoarthrosis-type non-union and refractures are common.

Various classification systems like those of Boyd, Crawford, and Anderson generally emphasize untreated bone radiographic findings, including the presence of fracture at birth and the involvement of fibula. But none of the above provides specific guidance for disease management or its results [15].

Treatment management in CPT

CPT seems not to be a homogenous entity but comprises several diseases with different prognoses [16,17]. The exact cause remains unclear, even if it is related to neurofibromatosis. The history of CPT is unpredictable and has no medical or surgical option, suggesting alterations in its natural history or pathophysiology. The bony union can be achieved by opting for surgery without ankle mortise for a stable foot, axial or rotational malalignment, and lower limb length equalization [18,19]. Treatment goals are followed by stabilization of the ankle mortise by fibular stabilization, osteosynthesis, and lower limb-length equalization. These goals are difficult to achieve, but the biological considerations remain the same [20]. One major problem is that refracture is often a complication after primary healing and may result in the reoccurrence of CPT. Hence, a safe and integral approach that is practical enough to ensure multiple treatment goals is needed and helps tackle CPT after surgical healing.

Ilizarov Fixation

**Figure 2:**
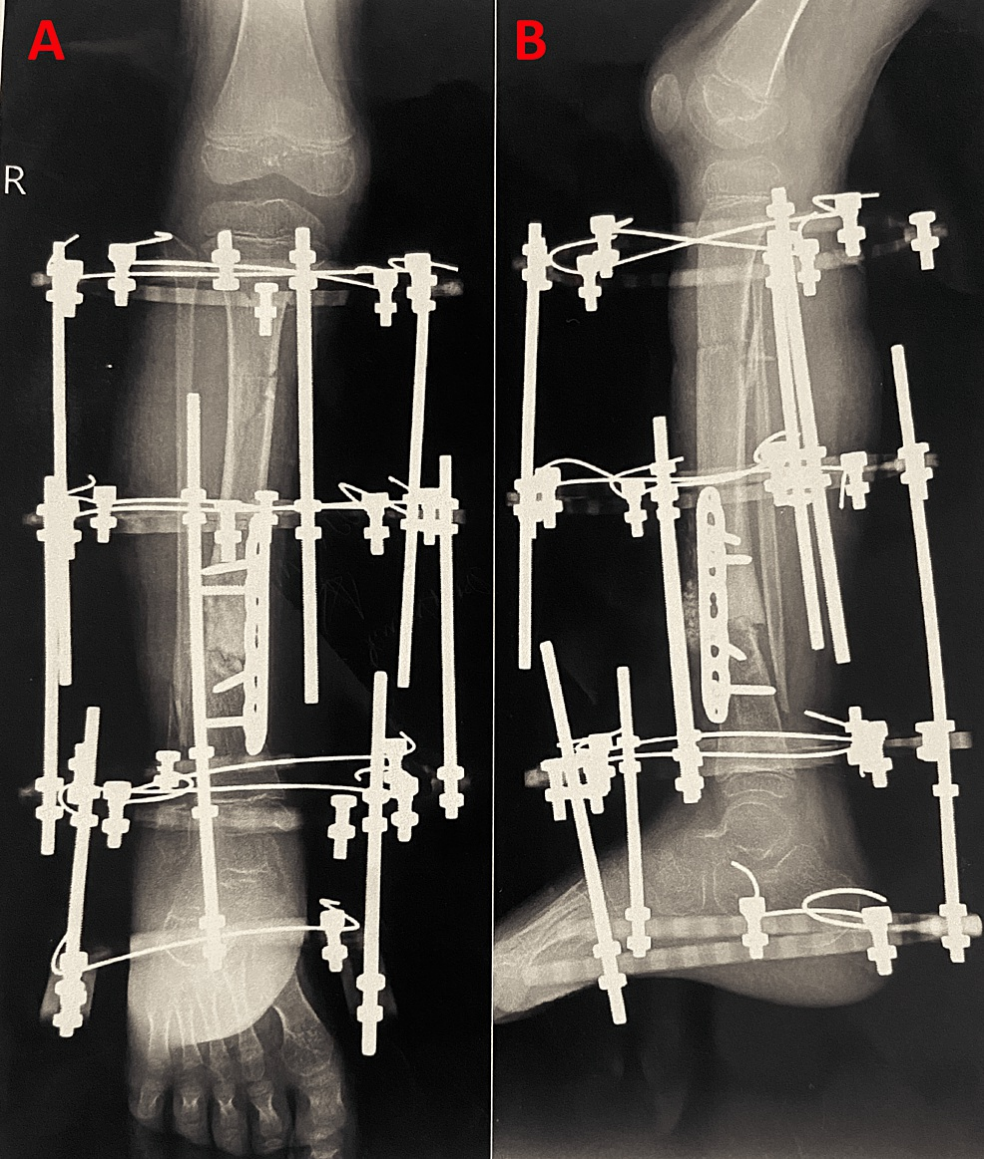
Radiograph showing Ilizarov fixation. Anterior-posterior and lateral radiographs that were taken postoperatively of a patient with fixators in Ilizarov fixation.

The Ilizarov method was introduced and represented in the western world during the late 19th century. It is accepted worldwide and popularized by surgeons for treating CPT as it addresses pseudoarthrosis and its related complex deformities. The basic concept behind Ilizarov fixation is that it involves gradually pulling the bones apart. This step is called distraction. It uses the body’s natural ability to generate and grow new bone between the surfaces pulled apart. This bony growth fills up the gap gradually, with the help of the Ilizarov fixator. Figure 2 shows Ilizarov's fixation.

Furthermore, in contrast to other treatment modalities, it doesn’t interfere with previous surgery and offers a great advantage of reapplication in case of refracture [20]. This technique provides the highest rate of bone fusion (75.5%) of pseudarthrosis and has a great success rate in correcting other deformities [21]. Post-operative management involves maintaining the Ilizarov ring fixator till fracture union, providing the patient with one dose of zoledronate, and repeating till the final fixator removal, which is basically for 3-5 months of post-op [22].

Sofield Fragmentation

It fragments bones into many pieces and then reshuffles and replants with intramedullary fixation.

Intramedullary Stabilization

It is introduced by Charnley. The intramedullary nail is one of the best methods for stabilizing pseudoarthrosis and is also used to prevent refracture, a major complication. Solid pins can be inserted rarely through knee fixed length and often in the heel, but the major drawback lies in replacement as the patient's age advances [11]. It is one of the innovative techniques of the “The Eiffel Tower” double titanium nails. Ilizarov fixation, transcutaneous electrical nerve stimulation (TENS) technique, bone grafting with less injury on metaphysis, early bone union, and early functional recovery [23].

Free Vascularized Fibral Grafts

Often, surgeons harvest contralateral fibula of length 10-12 cm along with intact vascular pedicles. Initially, this method includes simple screws and plating, but new intramedullary stabilization nailing methods have been introduced. Initially, these techniques include plating or simple screws, but more new intramedullary stabilization nailing methods have been introduced. The posterior and anterior tibial arteries and the associated veins are typically attached to the vascular pedicle. The primary and secondary unions of bone have a great success rate and have a distinct advantage over vascularised fibular grafts [11].

Radiographic and clinical evaluation methods

Main criteria for healing of CPT: A RUST (radiographic union scoring system for tibial fracture) score of more than 8 points indicates initial recovery [24]. The length of the tibia, which is from the midpoint of the proximal epiphyseal plate to the distal epiphysial plate, was measured using picture archiving and communication systems (PACS). Measurement of tibial valgus angle. PACS were used to measure the angle between the anatomical axis of the tibia and the epiphyseal line of the tibial proximal axis. If the tibial proximal axis valgus is more than 3, it is defined as tibial valgus. If the radiographic films suggest discontinuity of the bone cortex, it confirms that the refracture has occurred [25].

Complications

CPT being itself a complication involves many difficulties as well. Complications primarily related to using an external device include pin-track infection, neurovascular injury, loosening, breakage, axial deviation, osteopenia, and refracture at the pin insertion site. Valgus deformity and residual limb length discrepancy are usually reported with an overall complication rate of 30 to 1 [26,27]. Refracture is also one of the serious complications that may result in the re-establishment of pseudarthrosis. Mostly it continues to cause refracture because of dysplasia which mostly occurs in a subgroup of CPT causes.

Factors to be considered for minimizing the residual challenges

It is quite controversial at what age a surgeon should perform surgery. Some authors claimed that waiting for surgery until age 5-6 gives higher success rates [21]. Whereas some prefer surgery under 3-4 years [28,29]. Hence age can be considered a factor. Fibular stabilization is important to emphasize as deficient lateral fibular support causes ankle valgus deformity due to distal tibial wedging and fracture, resulting in immediate refracture [30]. Healing of the cross-sectional diameter of the tibia shows. Maximizing the cross-sectional area is a primary concern concerning healing at the level of pseudoarthrosis as it safeguards against refracture [31]. By using bone morphogenetic protein (BMP) in patients with CPT associated with neurofibromatosis type 1, hamartoma cells maintained some of the mesenchymal lineage phenotypes and didn't allow to change in the form of osteoblastic differentiation in response to BMP. Also, when compared to normal tibial cells, these cells were more osteogenic [32,33]. Based on this hypothesis, the fibrous hamartoma is resected completely during osteosynthesis. In past years it is observed that psychological changes along with their behavior after surgery influence patient post-op healing [34]. It is also seen that acceptance by society is an effective intervention for treating their psychological stress and anxiety [35].

## Conclusions

We have decoded, deciphered, and concluded CPT is a less frequently occurring orthopedic disease mainly involving children. Clinical features range from anterolateral bowing of the tibia to bone-related defects and complete bone non-union. Radiographic imaging includes hypertrophic or atrophic pseudoarthrosis and dystrophic or cystic lesions. Although CPT seems closely connected with type-1 neurofibromatosis, the exact pathogenesis is still unclear. The best treatment plan is surgery, including Ilizarov fixation and procedures like intramedullary stabilization, Sofield fragmentation, and free vascularized fibula grafts. Also, complications like refracture, pin-track infection, neurovascular injury, loosening, breakage, and axial deviation further complicate it for both surgeon and patient. The MSC treatment methodology can be combined to promote and enhance quick bone healing in patients with CPT. The quality of MSCs still in active research. A circular external fixator in combination with periosteal grafting is the ultimate and innovative method of treating CPT patients. Lot in recent years, the idea for the management of CPT has made a remarkable change by using rod constructs, MSC, or BMPs and hence shows guidance in bone deformity correction. Hence a precise combination of treatment options has to be considered by the surgeon to treat CPT. The age of the patient is also a factor of consideration for surgery.
